# Editorial: Reassessing Twenty Years of Vaccine Development against Tuberculosis

**DOI:** 10.3389/fimmu.2018.00180

**Published:** 2018-02-07

**Authors:** Ulrich E. Schaible, Stefan H. E. Kaufmann

**Affiliations:** ^1^Priority Program Area Infections, Department Cellular Microbiology, Forschungszentrum Borstel, Borstel, Germany; ^2^Department of Immunology, Max Planck Institute for Infection Biology, Berlin, Germany

**Keywords:** vaccines, tuberculosis, immune protection, global health, pulmonary

**Editorial on the Research Topic**

**Reassessing Twenty Years of Vaccine Development against Tuberculosis**

Tuberculosis remains the most threatening bacterial infection worldwide with 10.4 million infections and a death toll of 1.7 million people in 2016 according to World Health Organization (WHO) statistics (1). This makes tuberculosis the deadliest infectious disease of all. To those not familiar with this situation, this generally comes as a big surprise because drugs, diagnostics, and a vaccine are available as control measures. Currently, means of interventions, however, are insufficient, and better ones are urgently needed for early detection, successful treatment, and efficacious prevention.

20 years ago, in March 1997, the (WHO) had reported on the successful introduction of a novel therapeutic regiment, directly observed treatment, short course (DOTS). Compared with a 41% cure rate of other treatment programs, DOTS achieved a 77% rate. In a scenario, when tuberculosis was estimated to kill annually at least one-quarter of all patients worldwide, DOTS was therefore hailed as important milestone in tuberculosis control. It was hoped that the global tuberculosis epidemic could be controlled by the beginning of the twenty-first century, whereas otherwise an increase to nearly nine million annual cases by 2005 was projected. Indeed, numbers of deaths have been reduced by 2017; however, 10.8 million new tuberculosis cases in 2016, as well as the global increase in incidences of patients infected with multidrug resistant (MDR) strains spoiled the success story. Number of cases with MDR tuberculosis resistance against the two frontline drugs, such as rifampicin and isoniazid, tripled between 2009 and 2016 to 480,000 cases. Alarmingly, in several East European and Central Asian countries, the rate of MDR tuberculosis cases already exceeds 50% (1). Importantly, treatment of MDR patients comes with a significant economic burden to both the individual and the society (2). Despite the glimmer of hope provided by the recent introduction into the clinics of two new antimycobacterial drugs, such as Bedaquiline and Delamanid, patients with strains resistant to these new drugs have already been reported (3).

Consequently, the G20 Leader’s Declaration from June 2017 explicitly singled out MDR tuberculosis to be tackled with highest priority over other infections (4). This has led to the WHO Global Ministerial Conference on “Ending TB in Sustainable Development Era: A Multisectoral Response” in Moscow, November 16–17,^1^ which will be followed by The Stop TB Partnership Board Meeting in Delhi, March 2018.^2^ These two meetings are paving the way for the planned high level meeting of heads of state on tuberculosis to be held at the UN in New York, September 2018. Thus, tuberculosis has finally gotten on the agenda of political decision makers at highest level.

Tuberculosis is caused by members of the *Mycobacterium tuberculosis* complex, facultative intracellular bacteria able to thrive within otherwise potent innate defense cells, such as macrophages. More than 90% of all individuals infected with *M. tuberculosis* remain in a lifelong latent stage by immunity-mediated sequestering of the mycobacteria within granulomas. Reactivation of a latent infection occurs in the remaining ~10%, usually due severe immune suppressive conditions. Thus, in principle, human immunity is able to control—but likely not sterilely eliminates—the infection.

Unfortunately, the daunting race between the development of new therapeutic compounds and the evolution of resistance mutations will hardly produce a winner. This cycle can only be broken by enhancing population wide immune protection through a better vaccine. Immune protection of the only antituberculosis vaccine currently in use, *Mycobacterium bovis* bacillus Calmette–Guerin (BCG) is limited to severe disseminated primary infections in early childhood. By contrast, its protective efficacy against pulmonary tuberculosis in all age groups is dissatisfying, and geographically highly diverse with the tropical areas showing the lowest efficacy rates. Despite high worldwide vaccination coverage, the impact of BCG on the steep decrease of tuberculosis incidence rates in the developed world is therefore questionable and can rather be attributed to improved social, housing, and nutritional conditions, better health care, surveillance, and treatment systems. Consequently, the last 20 years saw tremendous efforts to improve vaccination strategies against tuberculosis.

For this Research Topic, we assembled experts to review and assess the current status of novel antituberculosis vaccine candidates, their efficacy, and prospects for implementation as well as the pitfalls and possible measures for improvement. Different rational approaches of vaccine design were followed in recent years, and the first novel vaccine candidates are currently being evaluated in clinical phases II and III, and initial results chastening the expectations. Therefore, a critical reassessment of all candidates is inevitable. The different strategies followed include genetically improved BCG strains, attenuated *M. tuberculosis* variants, recombinant viral vectors, and subunit vaccine candidates combined with novel more potent adjuvants. In addition, immune targets of novel vaccine types and immune enhancing strategies to improve vaccination efficacy have been identified.

In our Research Topic, Cardona summarizes how our current knowledge of the immune responses to *M. tuberculosis* can instruct vaccine design. The lessons learned from the current BCG vaccine and its future potential is detailed by Dockrell and Smith. The TBVAC2020 Consortium, consisting of a large number of European researchers and funded by the European Union, summarizes the different tuberculosis vaccine candidates, which they are developing and where they stand. This review also includes a comprehensive table of all current tuberculosis vaccine types studied. Nieuwenhuizen et al. discuss the current state of the promising recombinant BCG VPM1002, which has advanced into clinical phase III. Gonzalo-Asensio et al. review the attenuated vaccine strain MTBVAC, which is based on the deletion of two virulence-associated genes in a clinical *M. tuberculosis* isolate. The role of polyfunctional CD4+ T cells in immune protection against *M. tuberculosis* is summarized by Lewinsohn et al. To enhance vaccine efficacy, different strategies, which can modulate innate immunity during vaccination, are discussed by Schaible et al., whereas Lang and Schick look into how helminth infections affect antituberculosis immunity at the macrophage level.

In summary, we believe that this Research Topic provides an excellent overview on the current state of antituberculosis vaccine and corresponding research and the prospect of vaccine success and failure.

## Author Contributions

SK and US together wrote the Editorial.

## Conflict of Interest Statement

SHEK is coinventor of a vaccine against tuberculosis currently undergoing clinical trial testing. The authors declare that the research was conducted in the absence of any commercial or financial relationships that could be construed as a potential conflict of interest.

## Footnotes

^1^http://www.who.int/tb/features_archive/Online_Consultation_MinisterialConferenceDeclaration/en/.

^2^www.stoptb.org.
